# IDENTIFICATION OF SPASTIC MUSCLES INVOLVED IN ABNORMAL JOINT POSTURE IN PATIENTS WITH UPPER MOTOR NEURON SYNDROME: A NARRATIVE REVIEW

**DOI:** 10.2340/jrm.v58.45077

**Published:** 2026-07-02

**Authors:** Francois GENÊT, Vincent T. CARPENTIER, Guillaume CHAMBINAUD, Alberto ESQUENAZI, Marjorie SALGA

**Affiliations:** 1 UPOH (Unité Péri Opératoire du Handicap, Perioperative Disability Unit), Department of PMR “Suivi au long cours”, Raymond-Poincaré Hospital, GHU Paris-Saclay, Assistance Publique – Hôpitaux de Paris (AP-HP), Garches; 2 Versailles Saint-Quentin-en-Yvelines University (UVSQ – Paris Saclay University), UR 20262 Handistart, UFR Simone Veil Santé, Montigny le Bretonneux; 3 Department of Physical Medicine and Rehabilitation, Meulan – Les Mureaux Hospital, GHT Yvelines Nord, Les Mureaux, France; 4 Jefferson Moss-Magee Rehabilitation, Elkins Park, PA, USA

**Keywords:** spasticity, botulinum toxin, upper motor neuron syndrome, abnormal joint posture, motor nerve blocks, dynamic EMG, functional anatomy

## Abstract

Few studies have specifically investigated which muscles are involved in abnormal joint posture (AJP) due to muscle spasticity and should therefore be targeted for botulinum toxin injections. This gap has significant implications for treatment efficiency, safety, health economics, and sustainable healthcare. A 2000 to 2025 (July) PubMed search identified 3,488 articles, but only 7 articles met the criteria for providing a method to determine the muscles involved in AJP due to muscle spasticity. Of these, just 2 have proposed how to measure each muscle contribution and only 1 focused on identifying the muscle actually responsible for the observed AJP. There are many strategies for determining the muscles involved in spasticity-related AJP, but they are primarily based on inference. They draw on clinical skills, which incorporate descriptive and functional anatomy, knowledge of different muscle and joint structures, simple rules of biomechanics, determination of the exact phase of the movement involved, consideration of compensatory AJP in these motor control deficient patterns, and, of course, the patient’s goals. Achieving the authors’ proposed objective would enable the standardization of clinical practices, confirm the effectiveness of treatments for spasticity, particularly botulinum toxin, and ensure that the correct dose is injected in the right muscle.

## LAY ABSTRACT

People who have sustained brain or spinal cord injuries are paralyzed but also have abnormal joint posture due to muscle spasticity, causing their muscles to contract in an abnormal manner. Treatments, particularly botulinum toxin, can control these contractions by reducing the force generated by the affected muscles. The purpose of this paper is to highlight the lack of an identified method for demonstrating the involvement of a muscle in a given abnormal joint posture, to identify the clinical tools needed to identify them, and to propose a strategy for selecting the target muscle(s).

Scientific discourse frequently addresses the efficacy, safety, injection modalities (e.g., targeting and guidance), and dosage of botulinum toxin, which remains the primary intervention for spasticity management (1–10). However, there is a notable paucity of literature proposing decision-making frameworks or systematic methods for identifying the specific hypertonic muscles responsible for abnormal joint postures (AJP) (11–21). This gap may be attributed to the reliance on non-standardized expert opinions, which complicates the integration of these practices into evidence-based medical models (22).

Within a comprehensive care framework, inaccurate muscle targeting undermines the entire post-injection rehabilitation plan, resulting in significant repercussions for patient- and family-centred treatment goals (23). While clinicians often employ individualized approaches, a standardized practice has yet to be established (7, 24–27). The absence of a well-defined paradigm potentially obscures the clinical efficacy of botulinum toxin, particularly when injections are directed at muscles that do not contribute to the observed posture (28). From both an ethical and clinical optimization perspective, and considering the risk of complications (29), it is essential to develop a validated methodology to accurately identify target muscles. Such a method should integrate biomechanical analysis, functional anatomy, and the differentiation of contributing factors – such as muscle activation, co-activation, and contracture – to ensure a precise and effective injection protocol (15).

Scientific articles debate the efficacy, safety, injection modalities (targeting, guidance), doses to be administered, and other factors related to botulinum toxin, the first-line treatment for the management of spasticity (1–10). Surprisingly, there are hardly any studies proposing decision trees or methods for identifying problematic hypertonic muscle(s) to be injected for a given abnormal joint posture (AJP) due to muscle spasticity (11–21). This lack of scientific literature can be explained by the fact that the data in question are derived primarily from expert opinion regarding treatment modalities that have not yet been standardized, making it difficult to incorporate them into an evidence-based medical care model (22). Considering the global approach of this care, an injection target error has an impact on the entire post-injection care plan, particularly rehabilitation, with major repercussions on the patient and family treatment objective (23). Each clinician has thoughts on the subject, and no standardization of practices has yet been proposed (7, 24–27). The fact that this care paradigm currently does not have a well-defined method is one of the factors that can call into question the true efficacy of treatment interventions such as botulinum toxin (injection targeted at a muscle not responsible for or contributing to the posture) (28). From an ethical and optimization of care delivery point of view, given the potential complications of this type of treatment (29), it seems important to consider a validated method that can determine the true target muscles of a given AJP due to muscle spasticity, considering the multiple potential sources of error (muscle activation, co-activation, or contracture), biomechanical tools, and descriptive and functional anatomy useful to the clinician in developing a relevant injection scheme (15).

## METHODS

A systematic search was conducted in the PubMed database for articles published between January 2000 and July 2025 that detailed methodologies for identifying specific muscles involved in spastic deformities. The search strategy utilized the term “spasticity” in combination with “adult” and each major limb joint (“shoulder”, “elbow”, “wrist”, “hand”, “hip”, “knee”, “ankle”, and “foot”). Articles were excluded if the title or abstract failed to explicitly mention the subject of interest.

The initial search yielded 3,488 results. Of these, 168 articles required full-text review due to ambiguous abstracts. Following a targeted evaluation for muscle selection methods in spasticity-related deformities, 8 articles were retained (13–19, 21). Six of these studies described global “spastic patterns” across muscle groups but lacked strategies to verify individual muscle involvement or account for case-by-case variability (14–18, 21). Only 2 studies proposed methods to quantify the degree of involvement for each muscle within a pattern (13, 19). Ultimately, only 1 study established a definitive method for determining the specific muscles responsible for an observed clinical deformity (13).

### Gaps and controversies

Several sources of error have been identified as hampering the analysis of AJP due to muscle spasticity.

*Being lured by a “mirage”.* The clinician is often tempted to incriminate the most superficial muscles, as they are accessible by palpation and observation. In fact, it is easy to overestimate them, compared with deeper muscles whose expression of muscle overactivity is hidden from view. For example, it is easier to suspect biceps brachii than brachialis when looking at the elbow flexion deformity (13).

*Relying on what everyone else is doing.* Medical education is frequently predicated on observational learning and emulation, whereby students adopt the clinical reasoning and practices of their mentors, particularly when supporting literature is scarce. However, entrenched habits or a lack of training in contemporary diagnostic modalities may lead mentors to perpetuate suboptimal techniques. While mentorship is generally effective, the absence of validated methodologies for specific indications can result in the intergenerational transmission of clinical misinterpretations.

Furthermore, academic literature may introduce bias if efficacy is evaluated solely within the context of muscles approved by local regulatory agencies. Observational “real-world” studies highlight significant heterogeneity in injection practices; specific muscles are often prioritized based on regional authorizations rather than purely clinical requirements, leading to variations in the management of established deformities (30).

*How do we examine our patients?* Typically, a patient’s primary concern is functional in nature. Given that the manifestation of muscle overactivity frequently fluctuates across different functional contexts, the analysis of abnormal joint postures (AJP) must be conducted under realistic, stress-inducing conditions – specifically during movement and under muscle loading (closed chain) – rather than exclusively in a supine, non-weightbearing position (open chain). For instance, specific spastic upper limb patterns may shift significantly between a seated position and gait, or during the execution of targeted physical efforts. This variability in clinical presentation necessitates a personalized diagnostic approach, prompting researchers to advocate for the use of Goal Attainment Scaling (GAS) to evaluate the efficacy of spasticity interventions, such as botulinum toxin type A (26, 31, 32).

Understanding functional anatomy enables the clinician to better recognize the muscular interactions that may exist, depending not only on the sequence of movement, but also on the position of distal or proximal joints. Clinicians accustomed to evaluating these sequences use the properties of the tenodesis effect to determine whether a polyarticular muscle is involved in an AJP (20). For example, shoulder position in extension at the glenohumeral joint during the analysis of elbow flexion can be used to determine the role of the biceps brachii, particularly the long head, in limiting elbow extension. The supination component of the biceps brachii can change, becoming pronator in pronounced elbow flexion. The examiner must therefore test the biceps in different shoulder and forearm positions. The same principle applies to polyarticular muscles around other joints in the body.

**Fig. 1:**
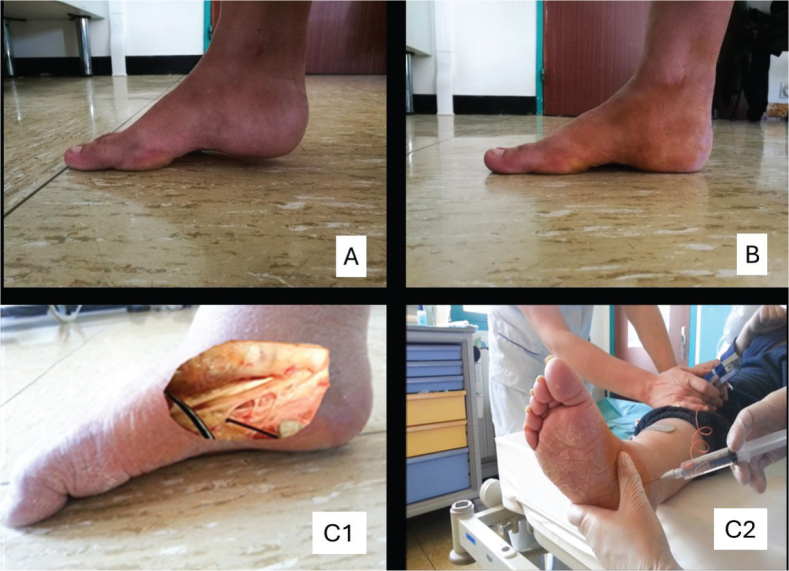
(A) Spastic Forefoot Equinus that has (B) been resolved after (C1, 2) a motor nerve branch block of the abductor hallucis.

**Fig. 2:**
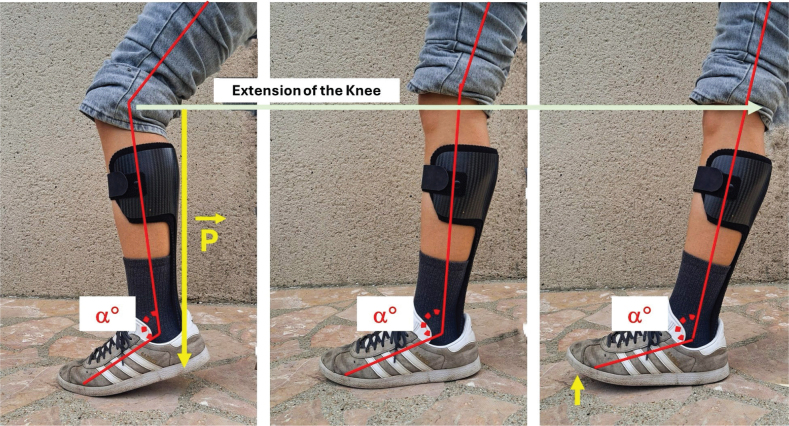
Ground effect: an equinus of the ankle induces knee extension as soon as weight is put on it, by seeking to place the heel on the ground. Filling the equinus induces knee flexion.

Keen observation is also essential: a heel that does not touch the ground is not always due to a hindfoot equinus. Indeed, forefoot equinus can also be a contributor. It may be due to prevalence or overactivity of the verticalizing muscles of the first metatarsal, which include: flexor hallucis longus, extensor hallucis longus, fibularis longus, and the intrinsics of the foot: abductor hallucis, flexor hallucis brevis, and adductor hallucis (Fig. 1). Observation during function, combined with detailed clinical examination, can detect this forefoot equinus associated with a 90° talo-tibial angle, avoiding the almost systematic injection of the triceps surae, which is a powerful knee extensor in a closed chain (foot on the ground) for these patient population who often have calf weakness (Fig. 2).

*Distinguishing between contracture and muscle overactivity.* Drugs used to treat muscle overactivity exert a paralyzing action on the muscle, focusing either on the nerve (surgical, chemical, or thermal neurolysis) or the motor endplate (botulinum toxin). Their functional efficacy therefore depends on maintaining the muscle’s contractile capacity. However, it has long been demonstrated that a muscle that does not receive physiological neurological information, as is the case in the UMNS, alters its structure and intrinsic characteristics (33, 34). Over time, some of these muscles lose their contractile capacity, becoming hypo-extensible or contracted (33–35). The loss of this contractile component means that the drugs used to paralyze the muscle are ineffective. Complementary treatment must therefore be considered (e.g., stretching in the case of hypo-extensibility; surgical lengthening in the case of contracture) (36, 37). The use of diagnostic nerve blocks with short-acting local anaesthetics makes it possible to distinguish between muscle overactivity and contracture (13, 38). Some of the muscles involved in a deformity may therefore be purely overactive, purely hypo-extensible or contracted, or be afflicted by a combination of these conditions. It is also essential to distinguish among each spastic muscle’s contribution to an AJP, to be able to set the right therapeutic approach and derive optimal treatment efficacy.

### Biomechanical dimension

*Lever arms and joint axes.* Experienced injectors apply biomechanical principles to analyse the pathophysiology of AJP. By mastering the understanding of the 3 types of lever arms, practitioners can determine if a specific muscle is exacerbating an AJP and evaluate its deforming potential. Furthermore, one must account for muscle length–tension relationships relative to the observed range of motion as muscles’ force production varies significantly based on the specific joint angle.

Reasoning in 3-dimensional space is essential, not only due to muscular physiology but also because articular axes – which guide movement in AJP – rarely align with standard anatomical planes. For instance, the scapula’s orientation on the rib cage shifts the glenohumeral joint axis 30° forward. Consequently, a “sagittal” shoulder flexion is actually a combination of flexion and 30° abduction. Understanding these functional ranges and the specific mechanics of joint types (e.g., ball-and-socket, hinge, or saddle) is indispensable for accurately analysing muscular action and degrees of freedom.

*“Open” or “closed” muscle chain?* Understanding “open-chain” and “closed-chain” biomechanics is critical to determining the impact of AJP in any given circumstance (39). Walking, for example, shifts lower-limb muscle physiology from an open-chain state during the swing phase to a closed-chain (weightbearing) state during the stance phase, which alters the segment’s force generation point.

Specifically, while the quadriceps are the sole knee extensors in an open chain (foot off the ground), 3 powerful muscle groups contribute to knee extension in a closed chain: the quadriceps, the gluteus maximus (GM), and the triceps surae (TS) (14, 15). In this weightbearing state, the GM and TS pull the femur and tibia backwards, respectively, to generate knee extension. This perspective helps clarify the functional roles of the GM and TS, which are often oversimplified in descriptive anatomy as merely a hip extensor and a plantar flexor based on open-chain observations.

*Type of muscle contraction: static, concentric, or eccentric.* The type of preferential work performed by certain muscles is often given limited consideration, and the impact this may have on function is therefore underestimated. It is important to review before implementing a treatment strategy. For example, weakness of the triceps surae considerably hinders the propulsion phase (concentric closed-chain work) during walking, but also affects the deceleration phase, and therefore affects standing balance (eccentric closed-chain work).

*Stress-dependent, useful, and pathological compensatory “toxic” deformations.* It is important to remember that a person with spasticity is first and foremost a patient presenting with a UMNS with motor control deficit (paresis). Functional anatomy enables us to better understand the potential benefits of muscle overactivity in compensating for a motor control deficit, or in achieving joint stability. This notion of “useful” AJP, which can easily be exemplified when we observe knee recurvatum in a patient with a knee extension muscle deficit in the closed-chain, *de facto* elicits the concept of a pathological compensatory “toxic” deformity when recurvatum increases over time, inducing injury to the joint and ligamentous structure, with pain and increase in energy consumption during walking (deforming kinetics increase with the amplitude of this recurvatum). The aim is therefore to reduce this recurvatum, but not to completely eliminate it, as the deformity compensates for the knee extensor muscle deficit. Similarly, an equinus foot that is not fully compensated with the use of a heel lift induces extension forces at the knee in the closed chain phase (14) (see Fig. 1). In analysing the factors that can contribute to AJP due to muscle spasticity, it seems appropriate also to consider the impact it may have in a given joint.

*Analysis in instrumented movement assessments.* For a long time, the thought was that instrumented movement analysis tools could replace clinical approaches. However, even as standardization efforts have been made by the societies concerned (40, 41), it became clear that these assessments are complementary, given the differences in clinical diagnosis, presentations, and reasoning. In fact, these analyses consider “normal” or physiological movement as a reference, with the aim of comparing the muscular activation or kinematics of a spastic patient with a normal data set (42). The quest for normalization or symmetry reflected by these tools is not ideal for a patient who uses compensatory movements or postures in order to adopt strategies for safe and efficient movement. The complementary approach of an experienced clinician, after an in-depth review of the kinematic data and other information, makes it possible to discuss whether the abnormal muscle activation is a primary problem to be treated or a compensation (useful spasticity, useful AJP) utilized to achieve a safer and more effective gait pattern. Instrumental analyses are still powerful tools with useful quantitative data, but they must consider the patient’s clinical condition and must be collected and interpreted with care by experienced clinical teams (37, 39, 43).

### Anatomical structural dimension: muscle shortening structure and modalities

*Muscle fibre orientation predicts muscle length during contraction.* Muscle-fibre orientation, particularly pennation, provides critical insights into contractile characteristics, force production capacity, and the resulting joint range of motion (44). Muscles characterized by circular fibre arrangements and limited contractile excursion are frequently less impacted by structural deformations that impose significant kinematic restrictions.

Given the substantial morphological variations among muscle architectures (e.g., unipennate, bipinnate, multipennate, convergent, and parallel), these structural traits significantly influence clinical interventions. Specifically, they dictate injection strategies aimed at optimizing toxin diffusion towards the motor endplates.

Emerging imaging modalities, such as ultrasound and elastography, offer promising avenues to validate current hypotheses regarding intramuscular diffusion. Furthermore, intrinsic variables – including muscle volume, fibre type distribution (e.g., Type I, Type II), and intended torque production – must be rigorously accounted for.

Finally, while the physiology of polyarticular muscles remains partially elusive, their architectural design suggests they function primarily as stabilizers and movement regulators, possessing significantly lower power generation than their mono-articular counterparts.

*Insertion modes: tendinous, aponeurotic.* Knowledge of muscle insertion provides important information on their force production, their angular performance sector and the risk of injury. A mono-articular muscle that inserts via an aponeurotic blade directly onto the bone, and which resembles a pulley like the brachialis, can develop greater force than the polyarticular biceps brachii, with its long, slender tendon.

### Reasoning: a simple, effective strategy

*Which muscles are agonist and antagonist to the deformity?* A muscle’s physiology can be determined in relation to the joint axis in all 3 planes of space (14). For example, any muscle or tendon pathway passing in the frontal plane behind the ankle’s bi-malleolar flexion/extension axis is an ankle plantar flexor, whereas if it passes anteriorly, it is a dorsal flexor. Any muscle whose muscular path passes medial to the vertical axis of the ankle and foot is an adductor, whereas if it passes lateral, it is an abductor. Finally, any muscle passing medial to the longitudinal axis of the foot is a supinator, while any muscle-passing lateral to the sagittal axis is a pronator.

**Fig. 3:**
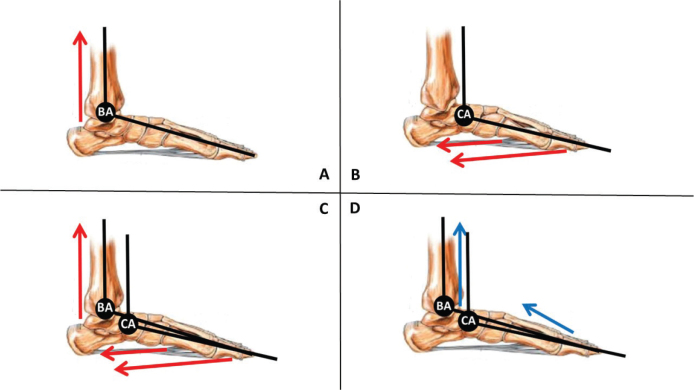
Clinical strategy for determining muscle targets. Example for the equinus foot. (A) Pure equinus of the hindfoot (extrinsic, behind the transverse bimalleolar axis [BA]): soleus and gastrocnemius. (B) Pure equinus of the forefoot (intrinsic, no tenodesis effect mobilizing the ankle). Below the transverse axis of Chopart’s joint: flexor digitorum brevis, flexor hallucis brevis, abductor hallucis, abductor digiti minimi, adductor hallucis, quadratus plantae, interosseous and lumbricals. (C) Rear and forefoot equinus (mixed, tenodesis effect mobilizing the ankle). Behind the transverse bimalleolar axis (BA) and below the transverse Chopart’s articular axis (CA): tibialis posterior, flexor digitorum longus, flexor hallucis longus, fibularis longus, fibularis brevis. Muscles participating in pure equinus of the rear foot and pure equinus of the forefoot can be involved but not alone. (D) Dorsiflexors of the ankle and the foot (extrinsic, anterior to the transverse bimalleolar axis [BA] and intrinsic: above Chopart’s transverse articular axis [CA]): extensor digitorum brevis and extensor hallucis brevis.

A muscle’s path around each of these joint’s axes enables us to determine its physiology in the 3 planes of space and thus understand its combined actions (Fig. 3). For example, in an open chain, tibialis posterior passes behind the frontal axis, making it a plantar flexor, inside the vertical axis, making it a foot adductor, and inside the sagittal axis, making it a supinator. If we look at the fibularis longus muscle, its path runs from the proximal and lateral part of the fibula to the lateral part of the head of the first metatarsal, passing retro-malleolar to the fibula and crossing under the foot from a tubercle on the fifth metatarsal. In effect, this muscle is an ankle plantar flexor, abductor, and foot pronator, but also verticalizes the first metatarsal (i.e., lowering). In open-chain spastic swing phase of gait, the forefoot may “wander in and out”. Verticalization of the first metatarsal means that the plantar surface of the hallux metatarsophalangeal joint is the first to be placed on the ground, before the others, leading to a *de facto* outward tilting of the foot when the other joints are placed on the ground under load, resulting in a sensation of lateral ankle instability. Sometimes a muscle antagonist can become agonist to an AJP if its tendon crosses the joint axis because of severe joint deformity. For example, the extensor carpi ulnaris can become a wrist flexor in case of severe flexion of the wrist, placing its tendon anterior of the flexion–extension axis.

**Fig. 4:**
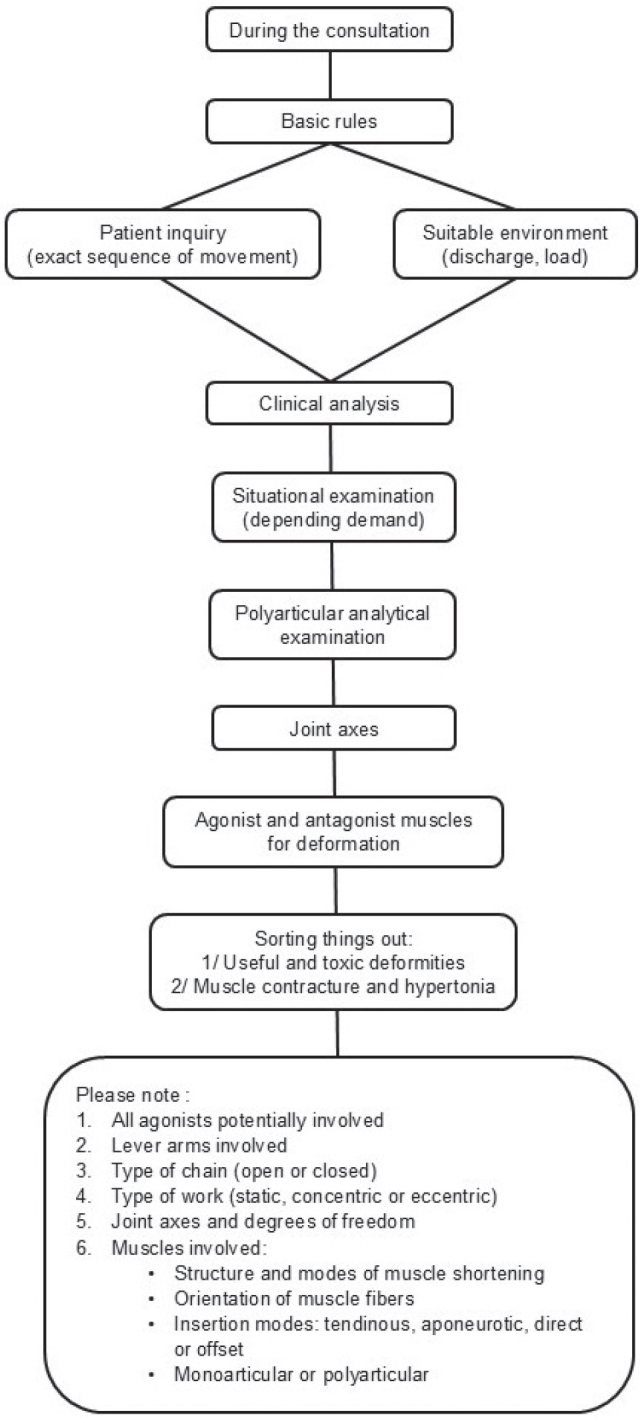
Decision-making flowchart during consultation.

*Taking all agonists into account.* While research examining the efficacy of botulinum toxin through joint range measurements occasionally yields statistically significant findings, the clinical relevance of such improvements often remains difficult to substantiate. In the management of equinus foot, the triceps surae are frequently prioritized for injection. However, achieving a substantial increase in ankle dorsiflexion may be precluded if auxiliary plantar flexors – such as the tibialis posterior, fibularis longus and brevis, and the long toe flexors – are neglected (see Fig. 2). Consequently, a systematic evaluation of all muscles potentially contributing to the deformity is essential to optimize therapeutic outcomes (14).

*A proposed diagnostic path.* Considering all these parameters, which are of importance in understanding the mechanisms of UMNS-related AJP, we propose a reasoning strategy to achieve the major objective for the effective treatment of muscle overactivity. Certain prerequisites are essential, and form part of the knowledge base before considering any treatment of AJP: (*i*) knowledge of descriptive anatomy and the different dimensions of functional anatomy, particularly in a closed chain; (*ii*) review of sources and knowledge (student must verify the dimensions taught and increase the number of mentors, in particular by calling on experts); (*iii*) ensure a critical analysis of the literature (the context in which the authors work, and in particular the muscle injection approval in the country concerned, and the method used to select target muscles for a given deformity must be described in detail in each study); and (*iv*) regular practice (as with any technical skill, expertise comes with practice). In clinical practice, we propose a diagnostic pathway, which attempts to take into account the elements described above, in order to select the muscle(s) truly responsible for AJP (see Fig. 2).

In conclusion, identifying the primary muscles contributing to abnormal joint posture (AJP) resulting from spasticity facilitates the establishment of a standardized clinical pathway. This systematic approach ensures precise identification of the muscles responsible for AJP, thereby aligning interventions with patient-specific therapeutic objectives. By precluding the injection of uninvolved musculature, this methodology mitigates the risk of adverse effects while enhancing the efficacy of targeted treatments.

Comprehensive proficiency in descriptive and functional anatomy, integrated with a focused clinical examination, remains fundamental to the management of spasticity. Despite rapid medical advancements, certain investigative domains continue to rely on foundational knowledge, systematic observation, and ecological clinical assessments. Standardizing these practices allows for a more rigorous evaluation of the treatment of AJP related to Upper Motor Neuron Syndrome (UMNS). Consequently, harmonizing specialized knowledge and pedagogical strategies regarding muscle identification in spasticity-induced AJP is essential for clinical care process progress.
